# Behavior Change Text Messages for Home Exercise Adherence in Knee Osteoarthritis: Randomized Trial

**DOI:** 10.2196/21749

**Published:** 2020-09-28

**Authors:** Kim Bennell, Rachel K Nelligan, Sarah Schwartz, Jessica Kasza, Alexander Kimp, Samuel JC Crofts, Rana S Hinman

**Affiliations:** 1 Department of Physiotherapy University of Melbourne Centre for Health, Exercise and Sports Medicine Carlton Australia; 2 Department of Epidemiology and Preventive Medicine Monash University Melbourne Australia; 3 Melbourne School of Population and Global Health University of Melbourne Centre for Epidemiology and Biostatistics Parkville Australia

**Keywords:** knee osteoarthritis, exercise, patient compliance, mobile phone, randomized controlled trial

## Abstract

**Background:**

Exercise is a core recommended treatment for knee osteoarthritis (OA), yet adherence declines, particularly following cessation of clinician supervision.

**Objective:**

This study aims to evaluate whether a 24-week SMS intervention improves adherence to unsupervised home exercise in people with knee OA and obesity compared with no SMS.

**Methods:**

A two-group superiority randomized controlled trial was performed in a community setting. Participants were people aged 50 years with knee OA and BMI ≥30 kg/m^2^ who had undertaken a 12-week physiotherapist-supervised exercise program as part of a preceding clinical trial. Both groups were asked to continue their home exercise program unsupervised three times per week for 24 weeks and were randomly allocated to a behavior change theory–informed, automated, semi-interactive SMS intervention addressing exercise barriers and facilitators or to control (no SMS). Primary outcomes were self-reported home exercise adherence at 24 weeks measured by the Exercise Adherence Rating Scale (EARS) Section B (0-24, higher number indicating greater adherence) and the number of days exercised in the past week (0-3). Secondary outcomes included self-rated adherence (numeric rating scale), knee pain, physical function, quality of life, global change, physical activity, self-efficacy, pain catastrophizing, and kinesiophobia.

**Results:**

A total of 110 participants (56 SMS group and 54 no SMS) were enrolled and 99 (90.0%) completed both primary outcomes (48/56, 86% SMS group and 51/54, 94% no SMS). At 24 weeks, the SMS group reported higher EARS scores (mean 16.5, SD 6.5 vs mean 13.3, SD 7.0; mean difference 3.1, 95% CI 0.8-5.5; *P*=.01) and more days exercised in the past week (mean 1.8, SD 1.2 vs mean 1.3, SD 1.2; mean difference 0.6, 95% CI 0.2-1.0; *P*=.01) than the control group. There was no evidence of between-group differences in secondary outcomes.

**Conclusions:**

An SMS program increased self-reported adherence to unsupervised home exercise in people with knee OA and obesity, although this did not translate into improved clinical outcomes.

**Trial Registration:**

Australian New Zealand Clinical Trials Registry 12617001243303; https://tinyurl.com/y2ud7on5

**International Registered Report Identifier (IRRID):**

RR2-10.1186/s12891-019-2801-z

## Introduction

### Background

Knee osteoarthritis (OA) is a global public health problem [1]. As OA has no cure, supporting patients to self-manage their condition is vital. Exercise is a core recommended treatment for knee OA [2,3] and is important for common comorbidities such as obesity, diabetes, and heart disease [4]. Exercise programs often involve initial supervision by a clinician, followed by unsupervised home exercise. Ideally, regular participation in exercise should be one of the long-term goals of self-management. Unfortunately, adherence to home exercise is often poor [5], particularly once clinician input ceases [6]. Numerous barriers can impact adherence, such as pain, negative beliefs about OA and exercise, and poor self-efficacy [7,8]. This decline in exercise adherence is typically mirrored by a gradual loss of initial clinical benefits [6,9]. Thus, scalable strategies to improve adherence to structured home exercise are thought to be important for better long-term patient outcomes [10].

There is uncertainty about how best to help people with knee OA adhere to exercise. Interventions that show promise include *booster* or *refresher* sessions with a physiotherapist and behavioral graded exercise, involving gradual increases in physical activity plus *booster* sessions [11]. However, ongoing clinician involvement may be unfeasible or impractical for many patients due to access challenges and/or cost. Instead, the use of digital communications such as SMS, email, or apps may be inexpensive and accessible options to help promote exercise adherence. As patients with knee OA tend to be older, SMS may have advantages over other forms of digital communication due to its widespread use, familiarity, and potential to overcome barriers related to device ownership (eg, not owning a smartphone) and access to and availability of Wi-Fi cellular data. The effectiveness of SMS-based interventions to promote healthy behaviors relevant to OA, such as physical activity, diet, and/or weight loss, has also been demonstrated in various settings and other conditions [12-14]. To date, the use of SMS to improve adherence to home exercise or physical activity in people with knee OA has only been evaluated in three pilot or feasibility studies [15-17].

### Objectives

The primary aim of the ADHERE randomized controlled trial (RCT) was to evaluate the effects of a theoretically informed 24-week SMS program [18] on self-reported adherence to a prescribed, unsupervised, structured home exercise program, undertaken after an initial 12-week period of physiotherapist supervision. We hypothesized that the SMS intervention would lead to greater exercise adherence than no SMS contact.

## Methods

### Trial Design

This parallel, two-arm superiority RCT is reported according to CONSORT (Consolidated Standards of Reporting Trials) [19], CONORT-EHEALTH (Consolidated Standards of Reporting Trials of Electronic and Mobile Health Applications and Online TeleHealth) [20], Template for Intervention Description and Replication (TIDieR) [21], and Consensus on Exercise Reporting Template (CERT) recommendations [22]. It was prospectively registered (Australian New Zealand Clinical Trials Registry #12617001243303), and the trial protocol is published [23]. Approval was obtained from the Institutional Human Research Ethics Committee (#1544919).

### Participants

This trial used participants completing another study, the TARGET trial [24], where participants visited a physiotherapist five times over 12 weeks for prescription of either a weight-bearing functional exercise program or a non–weight-bearing quadriceps strengthening exercise program. TARGET trial participants were recruited from the community in Melbourne, Australia, between September 2017 and May 2019 via advertisements through consumer organizations, social media, community locations, media, and our volunteer database. Inclusion criteria were as follows: (1) aged ≥50 years, (2) knee pain on most days of the past month, (3) knee pain for ≥3 months, (4) average overall pain severity ≥4 on an 11-point numeric rating scale (NRS), (5) tibiofemoral osteophytes on x-ray, (6) obesity (BMI ≥30 kg/m^2^), and (7) own a mobile phone with text messaging. The exclusion criteria are found in Multimedia Appendix 1.

The TARGET trial included face-to-face visits with members of the research team at the University of Melbourne. Only those who completed the TARGET trial final 12-week assessment and did not withdraw at this time point were enrolled into the ADHERE trial. Participants provided written informed consent to participate in the subsequent ADHERE trial at the time of TARGET trial enrollment.

### Randomization, Allocation Concealment, and Blinding

On completion of the TARGET trial final assessment (which served a dual purpose as the ADHERE trial baseline assessment), participants underwent 1:1 randomization into either SMS intervention or control (no SMS). Computer-generated randomization was prepared by the biostatistician (JK) in permuted blocks of sizes 6 to 12, stratified by type of exercise performed in TARGET and by exercise adherence at the final TARGET time point (0-1 sessions in the past week arbitrarily classified as *lower adherence* and 2-4 sessions *higher adherence*). Allocation was concealed in a password-protected computer program and accessed by a researcher not involved in enrollment or assessment. Participants were blinded to the study groups and to the study hypothesis through limited disclosure. They were informed at the TARGET trial enrollment that participation was for 9 months, with the initial 3 months comparing two exercise programs and the following 6 months investigating undisclosed adherence strategies, such as a logbook or text messages. To avoid influencing exercise adherence behavior, participants were not informed that two separate, but related, trials were being conducted or that they were being re-randomized into this trial. Outcome assessment was therefore blinded as the participants were deemed *assessors* in this RCT, given outcomes were participant reported. The statisticians were blinded to the group allocation.

### Interventions

All participants were asked to continue their allocated TARGET prescribed home exercise program unsupervised for 24 weeks [24] but to reduce the frequency from four times per week to three times per week (Multimedia Appendix 2). The frequency was reduced to facilitate adherence over the longer term while still meeting exercise guideline recommendations [25]. In the last appointment in the TARGET trial, the physiotherapists discussed with the participant the importance of progressing the exercises during the subsequent unsupervised phase (eg, by increasing resistance; changing stance surface; and/or varying the number of repetitions, direction, and speed of movement). Participants also received paper-based instructions for each exercise, including how to progress the exercise, and an optional logbook to record their exercise sessions if they wished.

#### SMS Intervention

Participants received a 24-week automated, semi-interactive SMS intervention delivered via mobile phone to support adherence to the home exercise program. The development of the SMS intervention was based on the Behavior Change Wheel framework [26] and is described elsewhere [18]. In brief, we identified key barriers or facilitators to exercise adherence in knee and hip OA and mapped these to the Theoretical Domains Framework [8]. Behavior change techniques linked to each barrier or facilitator [27] were then used to construct the content of the SMS messages.

**Figure 1:**
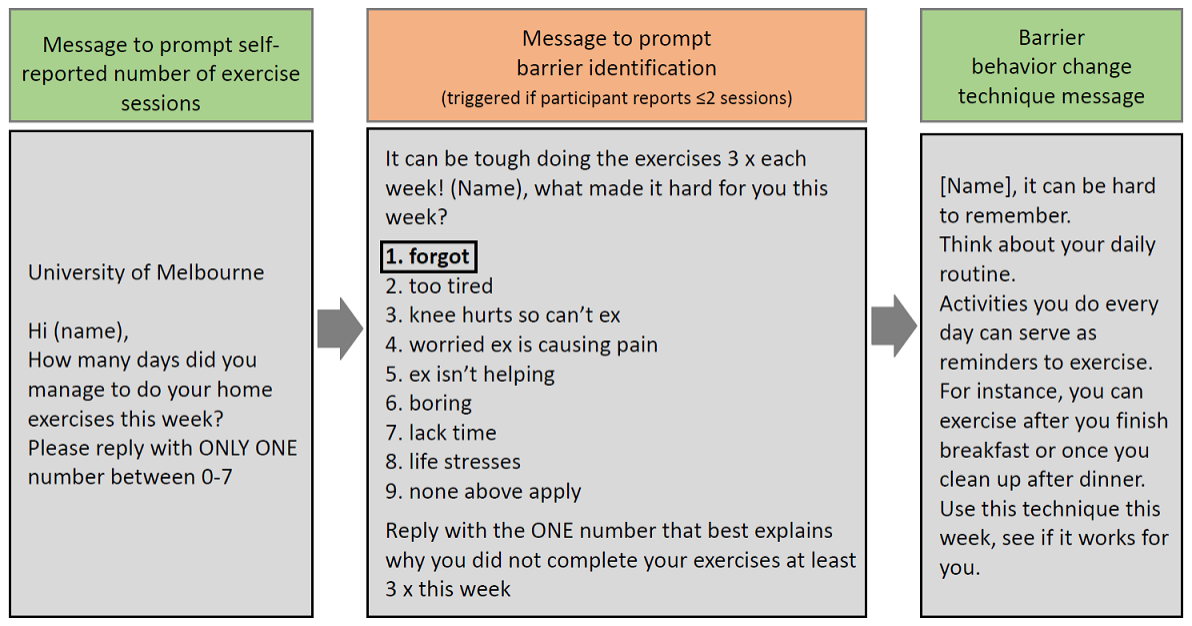
Example automated message sequence for a person with low exercise adherence and reporting their main barrier to exercise as “forgot.” Modified from the study by Nelligan et al 2019. Reproduced under the terms of the Creative Commons Attribution 4.0 license.

Participants received up to five text messages weekly, with message frequency reducing over the 24 weeks. Multimedia Appendices 3 and 4 [18] describe all message types and frequencies, whereas Multimedia Appendix 5 [18] outlines how the automated message sequence functioned. In summary, each week (weeks 1-8) to fortnight (weeks 9-24) participants received a message asking them to self-report the number of home exercise sessions completed in the previous week. Participants who completed ≤2 sessions then received a message prompting them to select their main reason (*barrier*) for not performing exercise sessions as prescribed (3 sessions per week) from a predetermined list (forgot, too tired, knee hurts so cannot exercise, worried exercise is causing pain, exercise is not helping, boring, lack of time, life stress, and none of the above apply to me). Barrier selection then triggered a message providing a suggestion tailored to address the selected barrier (example shown in Figure 1). Those who chose the barrier option of none of the above apply to me received a message encouraging them to continue exercising, but the message was not linked to a specific behavior change technique. Participants who reported being adherent (≥3 exercise session per week) received a positive reinforcement message. Program automation ensured that different messages were received each time. All participants, irrespective of their adherence, also received regular motivational SMS (twice weekly initially then once fortnightly by 24 weeks) containing suggestions linked to exercise facilitators. To enhance engagement, participants received special occasion messages (eg, birthday). Message lengths ranged from 105 to 420 characters, with literacy demands assessed as grade 5.4, well below the maximum eight-grade reading level recommended for consumer health care information [28].

#### Control—No SMS

Participants in the control group did not receive any SMS contact.

### Outcomes

Outcomes were self-reported and completed electronically (via REDCap) or on paper. The primary outcomes were two measures of adherence, collected at 24 weeks: (1) adherence to prescribed home exercise using the Exercise Adherence Rating Scale (EARS) Section B and (2) Number of days home exercises completed in the past week. EARS Section B has six items, each scored on a 5-point scale with terminal descriptors of *strongly agree* to *strongly disagree*. The total score ranges between 0 and 24, with higher scores indicating better adherence. This measure has acceptable internal consistency, high test-retest reliability (intraclass correlation coefficients [ICCs] from 0.91 to 0.97), and evidence of construct validity and responsiveness to change [29-31]. Participants were asked “In the past week, how many days did you do your recommended home exercises (maximum of 3 days)?” Response choices ranged from 0 to 3 days. Our test-retest reliability (2-week interval) with such a scale in 54 patients with knee OA was good (ICC [model 2,1]=0.79; 95% CI 0.66-0.87) with fair validity based on agreement with concealed accelerometer-measured session number (Spearman correlations from 0.26 to 0.48 over a 12-week period; method of accelerometer measure reported in the study by Nicolson et al [32]).

Secondary outcomes measured at baseline and 24 weeks, unless otherwise indicated, included the following: (1) adherence to home exercise program three times per week (24-weeks only) based on strength of agreement to the statement “I have been doing my exercise sessions 3 times each week as recommended” using an 11-point NRS with terminal descriptors *strongly disagree*=0 to *strongly agree*=10 [32]; (2) average overall knee pain in the past week using a NRS [33] with terminal descriptors of *no pain* (score=0) and *extreme pain* (score=10) [33]; (3) pain, other symptoms, function in daily living, function in sport and recreation, and knee-related quality of life in the last week using the Knee Injury and Osteoarthritis Outcome Score [34], ranging from 0 to 100, with higher scores indicating better outcomes; (4) health-related quality of life using Assessment of Quality of Life instrument [35] (version AQoL-6D), scores ranging from −0.04 to 1.00 and higher scores indicating better quality of life [35]; (5) Arthritis Self-Efficacy Scale, scores ranging from 0 to 10 and higher scores indicating greater self-efficacy [36]; (6) kinesiophobia using the Brief Fear of Movement Scale for OA, scores ranging from 6 to 24 and higher scores indicating greater kinesiophobia [37]; (7) Pain Catastrophizing Scale, scores ranging from 0 to 52 and higher scores indicating greater catastrophizing [38]; (8) Physical Activity Scale for the Elderly, scores ranging from 0 to >400 and higher scores representing greater physical activity [39]; and (9) participant-reported global overall change using a 7-point scale (terminal descriptors *much worse* to *much better*). Participants who reported *moderately better* and *much better* were classified as improved [40].

Adverse events (any problem participant believed was caused by advice received and required them to seek treatment or take medications and/or interfered with function for ≥2 days) were recorded via a questionnaire at 24 weeks. Medications and other knee OA treatments were recorded at 24 weeks using a customized survey.

Automatically collected SMS data included (1) number who opted to cease receiving messages, (2) mean (SD) number of SMS messages sent per participant, (3) mean (SD) participant reply rate to self-reported exercise sessions, (4) mean (SD) participant reply rate for barrier selection, and (5) group frequency of barriers selected.

### Sample Size

We conservatively estimated that 79.6% (102/128 of TARGET participants would be randomized into ADHERE, and of those, 80.3% (82/102) would be retained at week 24. We chose a moderate effect size of 0.6, given that smaller effects are unlikely to be clinically relevant [41]. With 40 participants per group, we would have 83% power to detect an effect size of 0.6 with a two-sided significance level of .05, assuming a correlation between baseline home exercise adherence and adherence outcomes at 24 weeks of 0.4, based on data from our previous trials [42-44], and including baseline adherence as a covariate in regression models.

### Statistical Methods

Analyses were performed by biostatisticians (JK and SC) using Stata (StataCorp, version 16) software and intention-to-treat. Baseline characteristics of participants who did and did not provide both primary outcomes were compared using *t* tests or chi-square tests. Missing outcomes were imputed using chained equations with predictive mean matching and five nearest neighbors for continuous outcomes and logistic regression imputation models for binary improvement outcomes. Data were imputed for each group separately. Imputation models for continuous outcomes at 24 weeks included all baseline and outcome variables, where appropriate. Imputation models for binary variables omitted all outcome variables because of the potential for perfect prediction, including only baseline variables. Estimates from 20 imputed data sets were combined using Rubin’s rules [45]. Standard diagnostic plots assessed the validity of model assumptions and imputed data sets. For the primary outcome of exercise adherence EARS Section B, the mean between-group difference at week 24 was estimated using a linear regression model adjusted for baseline measures and the stratifying variables of the TARGET exercise group and dichotomized baseline adherence. For the primary outcome of number of days home exercises completed in the past week and the secondary outcome of adherence to home exercise, the mean between-group difference at week 24 was estimated using linear regression models, adjusted only for the stratifying variables. For the continuous secondary outcomes, the mean between-group difference in change (baseline minus follow-up) at week 24 was estimated using linear regression models adjusted for baseline measures and stratifying variables. The proportion of participants with overall self-perceived improvement was compared between groups using a logistic regression model adjusted for stratifying variables, with results presented as odds ratios and risk ratios. Complete case analyses were also conducted.

## Results

### Participants

**Figure 2:**
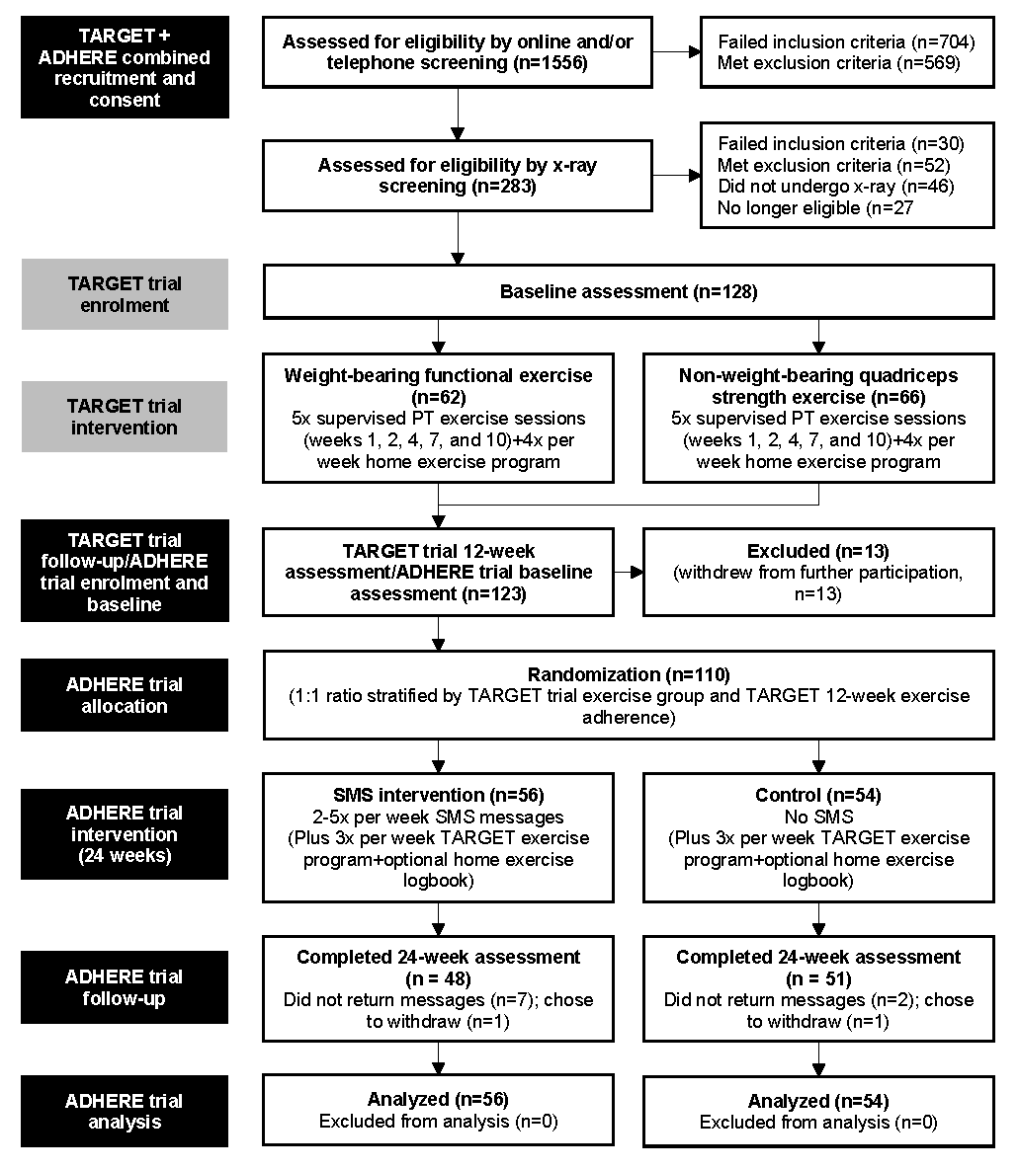
Flow diagram of preceding TARGET trial and ADHERE trial procedures. PT: physiotherapy.

We randomized 110 participants (56 SMS group and 54 no SMS), with 99 (90.0%) completing both primary outcome measures at week 24 (48/56, 86% SMS group and 51/54, 94% no SMS; Figure 2). The sample had a mean (SD) BMI of 37.3 (SD 6.4) kg/m^2^ and were predominantly female (74/110, 67.2%). Groups were comparable at baseline (Table 1). Participants who provided both primary outcomes were comparable with those who were missing at least one (Multimedia Appendix 6).

**Table 1:** Baseline characteristics of participants by group.

Characteristics		SMS (n=56)	Control (n=54)
Age (years), mean (SD)		61.7 (6.7)	62.9 (6.8)
Males, n (%)		21 (38)	15 (28)
Height (m), mean (SD)		166.1 (8.5)	165.4 (9)
Body mass (kg), mean (SD)		102.7 (18.6)	102.3 (18.3)
BMI (kg/m^2^), mean (SD)		37.3 (6.8)	37.4 (6)
**Radiographic disease severity KL^a^** **grade, n (%)**			
	2	9 (16)	11 (20)
	3	35 (63)	32 (59)
	4	12 (21)	11 (20)
Currently employed, n (%)		35 (63)	32 (59)
Symptom duration (years), mean (SD)		8.1 (6.9)	8.3 (8)
Unilateral symptoms, n (%)		13 (23)	11 (20)
**Problems in other joints, n (%)**		45 (80)	41 (76)
	Hand	22 (39)	20 (37)
	Neck	23 (41)	7 (13)
	Back	30 (54)	23 (43)
	Hip	20 (36)	17 (31)
	Foot	17 (30)	13 (24)
	Shoulder	17 (30)	10 (19)
**Treatments for knee OA^b^** **in the last 6 months, n (%)^c,d^**			
	At least one treatment	45 (80)	43 (80)
	Hot or cold treatment	15 (27)	17 (31)
	Shoe orthotics	4 (7)	9 (17)
	Massage	10 (18)	9 (17)
	Knee braces	6 (11)	3 (6)
	Hydrotherapy	5 (9)	6 (11)
	Manual therapy	4 (7)	2 (4)
	Walking stick	4 (7)	3 (6)
	Acupuncture	4 (7)	0 (0)
	Arthroscopic surgery	0 (0)	0 (0)
	Transcutaneous electrical nerve stimulation	1 (2)	0 (0)
	Ultrasound	0 (0)	0 (0)
	Injections	0 (0)	0 (0)
**Current pain medication use, n (%) ^c,d^**		39 (70)	40 (74)
	Nonsteroidal anti-inflammatories	20 (36)	23 (43)
	Cyclooxygenase-2 inhibitors	2 (4)	4 (7)
	Analgesics (paracetamol combinations)	31 (55)	34 (63)
	Topical anti-inflammatories	21 (38)	15 (28)
	Oral corticosteroids	0 (0)	0 (0)
	Oral opioids	3 (5)	0 (0)
**TARGET exercise group, n (%)**			
	Nonweight-bearing exercise	30 (54)	28 (52)
	Weight-bearing exercise	26 (46)	26 (48)
TARGET adherence to home exercise^e,f^, mean (SD)		18.1 (6.8)	17.8 (6.6)
TARGET no of days exercised in past week^f^, mean (SD)		2.8 (1.5)	2.4 (1.5)

^a^KL: Kellgren and Lawrence.

^b^OA: osteoarthritis.

^c^Defined as at least once per week in the prior month.

^d^Numbers do not add up to total as participants could choose more than one.

^e^Measured by the Exercise Adherence Rating Scale (EARS) Section B.

^f^Measured at week 12 in the TARGET trial.

A total of 17 participants reported adverse events (none serious), mostly increased knee pain or pain elsewhere (Multimedia Appendix 7). The use of pain medications and co-interventions was similar between groups (Multimedia Appendix 7).

In the SMS group, two participants chose to stop receiving messages. Over the 24 weeks, the mean (SD) number of SMS sent to each participant was 57.9 (SD 9.1) messages. The mean (SD) reply rate per participant to self-reporting home exercise sessions was 66% (SD 34%) and to selecting a barrier (if <3 exercise sessions reported) was 73% (SD 35%). Across the group, the most commonly chosen barrier was lack of time (n=44), followed by none apply (n=43), life stress (n=28), knee hurts so cannot exercise (n=16), worried exercise is causing pain (n=7), forgot (n=6), too tired (n=5), exercise is not helping (n=2), and exercise is boring (n=1).

### Primary Outcomes

Both primary outcomes provided evidence of greater home exercise adherence with the SMS intervention compared with control (Tables 2 and 3). At week 24, the SMS group reported higher scores on the EARS (mean 16.5, SD 6.5 vs mean 13.3, SD 7.0; mean difference 3.1, 95% CI 0.8-5.5; *P*=.01) and more days performing home exercise in the past week (mean 1.8, SD 1.2 vs mean 1.3, SD 1.2; mean difference 0.6, 95% CI 0.2-1.0; *P*=.01) than the control group. Specifically, in the SMS group, 23% (11/48) participants did not perform home exercises in the past week, 8% (4/48) performed home exercises on 1 day, 29% (14/48) on 2 days, and 40% (19/48) on 3 days in the past week. In the control group, 35% (18/51) participants did not perform home exercises in the past week, 20% (10/51) performed home exercises on 1 day, 22% (11/51) on 2 days, and 24% (12/51) on 3 days in the past week. Analyses using complete case data yielded similar results (Multimedia Appendix 8).

**Table 2:** Mean (SD) scores on continuous outcome measures across time, by group.

Outcomes		Baseline		24 weeks	
		SMS (n=56), mean (SD)	Control (n=54), mean (SD)	SMS (n=48)^a^, mean (SD)	Control (n=49)^b^, mean (SD)
**Primary outcomes**					
	Adherence to prescribed home exercise (EARS^c^ Section B)^d^	—^e^	—	16.3 (6.6)	13.4 (7.1)
	Number of days on which home exercises were completed in the past week^d^	—	—	1.9 (1.2)	1.3 (1.2)
**Secondary outcomes**					
	Adherence to home exercise thrice weekly (NRS)^d,f,g^	—	—	6.0 (3.8)	5.1 (3.7)
	Overall average knee pain (NRS)^h^	3.5 (2.1)	3.8 (2.4)	4.1 (2.2)	4.0 (2.3)
	Pain (KOOS)^i^	64.3 (14.9)	63.2 (19.8)	64.9 (17.3)	64.4 (20.1)
	Other symptoms (KOOS)	64.3 (17.0)	64.3 (17.4)	66.6 (18.5)	64.9 (18.3)
	Function (KOOS)	72.2 (15.6)	70.6 (20.7)	72.4 (17.6)	70.0 (21.1)
	Sport and recreation (KOOS)	33.4 (22.3)	39.5 (23.3)	37.3 (24.8)	41.6 (27.7)
	Knee-related quality of life (KOOS)	44.4 (19.9)	47.9 (21.7)	46.1 (22.0)	47.8 (23.0)
	Health-related quality of life (AQoL)^j^	0.76 (0.18)	0.81 (0.12)	0.77 (0.15)	0.78 (0.15)
	Self-efficacy: pain (ASES)^k^	6.6 (2.0)	6.9 (2.1)	6.6 (2.2)	6.4 (2.1)
	Self-efficacy: function (ASES)	8.4 (1.2)	7.8 (2.2)	8.3 (1.5)	8.2 (1.7)
	Self-efficacy: other (ASES)	7.2 (1.8)	7.5 (2.1)	7.4 (2.0)	7.6 (1.9)
	Kinesiophobia (BFOMS)^l^	12.5 (3.4)	12.2 (4.0)	12.1 (3.6)	12.1 (3.8)
	Pain catastrophizing (PCS)^m^	6.0 (7.7)	7.4 (9.9)	6.9 (9.6)	6.2 (7.1)
	Physical activity (PASE)^n^	176.7 (86.9)	173.9 (82.8)	190.5 (111.3)	174.0 (71.0)

^a^n=48 for both primary outcomes and n=45 for all secondary outcomes.

^b^n=49 for both primary outcomes and n=45 for sport and recreation (KOOS), AQoL, BFOMS, and PASE. n=46 for all other secondary outcomes.

^c^EARS: Exercise Adherence Rating Scale (0-24; higher scores indicate better adherence).

^d^Adherence data only collected at 24 weeks.

^e^Represents data about adherence that can only be collected at 24 weeks and not baseline.

^f^NRS: numeric rating scale.

^g^Adherence to home exercise thrice weekly—agreement with statement “I have been doing my exercise sessions 3 times each week as recommended” with responses collected using an 11-point NRS and terminal descriptors *strongly disagree*=0 to *strongly agree*=10.

^h^Overall average pain NRS (0-10; higher scores indicate worse pain).

^i^KOOS: Knee Injury and Osteoarthritis Outcome Score (0 to 100; lower scores indicate worse pain/symptoms/function/quality of life).

^j^AQoL: Assessment of Quality of Life instrument (−0.04 to 1.0; higher scores indicate better quality of life).

^k^ASES: Arthritis Self-Efficacy Scale (1-10; higher scores indicate better efficacy).

^l^BFOMS: Brief Fear of Movement Scale (6-24; higher scores indicate greater fear).

^m^PCS: Pain Catastrophizing Scale (0-52; higher scores indicate greater catastrophizing).

^n^PASE: Physical Activity Scale for the Elderly (0-400+; higher scores indicate greater activity).

**Table 3:** Mean (SD) scores at week 24 or mean (SD) change within groups, from baseline to week 24, and mean (95% CI) difference between groups (adjusted for baseline value of outcome, TARGET exercise group, and dichotomized baseline adherence), for continuous outcomes, using multiply imputed data.

Outcomes						SMS, mean (SD)		Control, mean (SD)		SMS−control^a^, mean difference (95% CI)		*P* value
**Mean (SD) at week 24** **and mean (95% CI) difference between groups**												
	**Primary outcomes**											
		Adherence to prescribed home exercise (EARS^b^ Section B)			16.5 (6.5)			13.3 (7.0)		3.1 (0.8 to 5.5)		.01
		Number of days on which home exercises were completed in the past week^c,d^			1.8 (1.2)			1.3 (1.2)		0.6 (0.2 to 1.0)		.01
	**Secondary outcomes**											
		Adherence to home exercise three times weekly (NRS)^c,e^			6.0 (3.8)			4.9 (3.7)		1.1 (−0.4 to 2.6)		.14
**Mean (SD) change within group (baseline minus week 24)** **and mean (95% CI) difference in change between groups**												
		Overall average knee pain (NRS)^d,f^			−0.6 (2.4)		−0.2 (2.2)		−0.2 (−1.1 to 0.6)		.59	
		Pain (KOOS)^g^			−0.8 (14.9)		−2.6 (14.1)		1.3 (−4.6 to 7.3)		.66	
			Other symptoms (KOOS)		−2.9 (17.3)		−1.7 (13.5)		−1.2 (−7.5 to 5.0)		.70	
		Function (KOOS)			−0.0 (18.5)		−0.5 (14.0)		−0.2 (−6.7 to 6.3)		.95	
		Sport and recreation (KOOS)			−3.5 (22.1)		−3.0 (21.9)		1.2 (−8.4 to 10.8)		.81	
		Knee-related quality of life (KOOS)			−2.2 (23.0)		−2.3 (16.2)		1.3 (−6.7 to 9.4)		.75	
		Health-related quality of life (AQoL)^h^			0.00 (0.13)		0.03 (0.15)		−0.01 (−0.06 to 0.04)		.68	
		Self-efficacy: pain (ASES)^i^			0.0 (2.1)		0.6 (2.6)		−0.4 (−1.2 to 0.4)		.35	
		Self-efficacy: function (ASES)			0.1 (1.7)		−0.4 (2.1)		0.1 (−0.5 to 0.7)		.78	
		Self-efficacy: other (ASES)			−0.0 (1.9)		−0.1 (2.3)		0.1 (−0.6 to 0.9)		.69	
		Kinesiophobia (BFOMS)^j,k^			0.4 (2.6)		−0.2 (3.6)		0.5 (−0.8 to 1.7)		.44	
		Pain catastrophizing (PCS)^k,l^			−1.9 (7.9)		0.9 (10.1)		−2.0 (−5.2 to 1.2)		.22	
		Physical activity (PASE)^m^			−15.1 (90.1)		0.9 (82.6)		−17.5 (−53.0 to 18.0)		.33	

^a^For mean difference between groups, positive differences favor SMS.

^b^EARS: Exercise Adherence Rating Scale (0-24; higher scores, better adherence).

^c^Not adjusted for baseline value of outcome.

^d^Adherence to home exercise thrice weekly: agreement with statement “I have been doing my exercise sessions 3 times each week as recommended” collected using an 11-point numeric rating scale (NRS) and terminal descriptors *strongly disagree*=0 to *strongly agree*=10.

^e^NRS: numeric rating scale.

^f^Overall average knee pain NRS (0-10; higher scores, worse pain).

^g^KOOS: Knee Injury and Osteoarthritis Outcome Score (0-100; lower scores, worse pain/symptoms/function/quality of life).

^h^AQoL: Assessment of Quality of Life instrument (−0.04 to 1.0; higher scores, better quality of life).

^i^ASES: Arthritis Self-Efficacy Scale (1-10; higher scores, better efficacy).

^j^BFOMS: Brief Fear of Movement Scale (6-24; higher scores indicate greater fear).

^k^For change within groups, positive changes indicate improvement.

^l^PCS: Pain Catastrophizing Scale (0-52; higher scores, greater catastrophizing).

^m^PASE: Physical Activity Scale for the Elderly (0-400+; higher scores, greater activity).

### Secondary Outcomes

There was no evidence of a between-group difference in adherence (NRS) at 24 weeks or in change in clinical outcomes (Tables 2 and 3). Within-group changes in both groups were relatively small (Tables 2 and 3). Similar proportions of participants in both groups reported global improvement overall since baseline (SMS 23/45, 51% vs control 19/46, 41%; odds ratios 1.48, 95% CI 0.61-3.57; *P*=.38; relative risk, 1.20, 95% CI 0.70-1.71; *P*=.39). Analyses using complete case data yielded similar results (Multimedia Appendix 9).

## Discussion

### Principal Findings

We found that an automated behavior change theory–informed, semi-interactive SMS intervention improved self-reported adherence to a prescribed unsupervised home-based exercise program over 24 weeks, evidenced by both primary outcomes, when compared with no SMS contact in people with knee OA and obesity. However, greater adherence to home exercise with SMS support did not translate into improvements in secondary clinical outcomes.

The greater exercise adherence may be linked to the rigorous development of the SMS program based on a widely used framework, the Behavior Change Wheel [26]. As our program incorporated several elements, we could not determine which elements were most effective in eliciting the desired exercise behavior. Regular receipt of a message asking to self-report exercise completion can act as a reminder, and self-monitoring behavior increases physical activity adherence in patients with OA [46] and in adults who are overweight or have obesity [47]. Our bank of 198 different text messages included 20 behavior change techniques targeting 13 modifiable barriers and 9 facilitators to exercise previously identified in people with OA [8]. As the SMS program was semipersonalized, the number and type of behavior change techniques employed differed across participants, depending on their adherence and barrier selection. However, in 28% of barrier replies, participants selected *none apply*, meaning that no targeted response message could be sent, which may have attenuated the benefits of the program.

Despite greater adherence to home exercise in the SMS group, we found no between-group difference in any secondary clinical outcome. Both groups either maintained or had slight diminution of the clinical improvements resulting from the preceding 12-week physiotherapist-supervised exercise phase (TARGET trial) [24]. This lack of further improvement may relate to inadequate exercise progression, including suboptimal intensities, during this unsupervised phase. Although it is presumed that there is a relationship between exercise adherence and outcomes in knee OA, surprisingly, the nature of the relationship is unclear given the limited research and contradictory findings [6,48-50]. For example, some studies using self-report adherence measures have found that greater adherence is linked with better pain and function outcomes [6,48,50]. In contrast, a study using concealed accelerometers to accurately assess adherence to a 12-week home strengthening program showed no evidence of an association [49]. It is possible that the greater adherence seen in our SMS group may have been of insufficient magnitude (between-group difference of 3.1 EARS units and 0.6 days exercised) to impact clinical outcomes, especially as adherence actually decreased in both groups from relatively high levels after the supervised exercise phase. This is supported by a recent study that showed a minimally important change of 5.5 units for the Brazilian Portuguese version of the EARS, albeit in people with chronic low back pain [30]. Another explanation relates to the multi-dimensional nature of adherence, which is not fully captured in our measures. For example, exercise regularity and intensity may be elements of the prescribed exercise program that may be important to clinical outcomes [51].

### How our Findings Compare With Those of Other Studies

Although numerous strategies are suggested to improve adherence to exercise in people with OA, many of which are clinician-centric such as *booster sessions*, there is limited RCT evidence available to inform clinical practice [11]. To our knowledge, only three pilot or feasibility studies have specifically investigated an SMS intervention aimed at improving exercise adherence in people with knee OA [15-17] and none measured adherence. In one study, short video messages providing visual prompts to home exercises were sent every second day for 6 weeks to 5 people [16]. Participants found the messages *very useful*, and although there was no difference in functional outcomes compared with a control group (n=9), the direction of improvement favored the intervention. In another study, 27 people received four activity-promoting text messages per week for 6 weeks to reinforce content from an OA educational booklet. Participants meaningfully engaged with the intervention (100% read messages and 89% replied), reporting it to be enjoyable and personally relevant. Improvements were seen in perceptions of exercise and pain [15]. In the third study, 19 people who had completed an education and exercise program (Good Life with Arthritis: Denmark) received text messages providing general physical activity advice, three times per week for 6 weeks, whereas the control group received no text messages (n=19) [17]. The results did not indicate any potentially beneficial effects of the intervention on physical inactivity or clinical outcomes. As we cannot draw conclusions about efficacy from these studies, we provide the first evidence from a fully powered RCT about the effectiveness of an SMS strategy on adherence to a home exercise program in people with knee OA. Our results also concur with other RCTs in healthy older adults [52] and in those with frozen shoulder [53], which found that automated SMS programs can improve self-reported adherence to home-based exercise.

### Strengths and Limitations of the Study

The strengths of our study include a systematically designed SMS intervention informed by behavior change theory; participant, and thereby assessor, blinding to group allocation; excellent retention at 24 weeks; and acceptable user engagement with the SMS program. There are some limitations to this study. First, accurate measurement of exercise adherence is challenging [54], and there is no gold standard. As is the case with most studies [54], we used self-reported exercise adherence measures to ensure feasibility, given our large sample and extended time frame. However, self-report measures are subjected to recall bias and typically overestimate [32] due to social desirability bias. This latter bias may be accentuated in the SMS group, given that it received regular reminders about the importance of exercise. Second, only those who completed the preceding TARGET study were enrolled as participants. This may have introduced selection bias, particularly by increasing the likelihood that a more adherent group was enrolled, which could make it more difficult to detect an effect of the SMS program on exercise adherence. However, this is less likely given that 90% of the original TARGET study participants took part. Third, we do not know whether the improved exercise adherence is sustainable over time once SMS contact ceases or whether our findings can be generalized to patients who may be less motivated than research volunteers or to those without obesity or generalized to a home exercise program that is unsupervised from its outset. Nonetheless, the characteristics of our sample broadly reflect those of the general knee OA patient population, which includes greater proportion of females, and people who are more likely to have overweight or obesity, and be of an older age [55].

### Implications for Clinical Practice and Future Research

Our results provide preliminary evidence that the use of SMS may promote patient adherence to a core recommended OA treatment (exercise), which is an important clinical priority [56]. Mobile phone text messaging programs are an increasingly popular method for delivering health behavior change interventions [12]. This is unsurprising given the ubiquitous use of mobile phones across all populations and age groups and the many benefits of using SMS technology such as convenience, instantaneous communication, cost-effectiveness [57], and user acceptability. Our SMS program is a scalable, inexpensive intervention that could be incorporated into existing or future web-based exercise resources and/or used by clinicians to enhance adherence of their patients to their own exercise prescription. Although we chose SMS as the delivery mode, the message content and program could be converted into formats suitable for delivery by other communication forms such as email or a mobile app. It could also be adapted for use in other health conditions where exercise is a core treatment and its effectiveness is tested. Further research into modification of the program and its implementation is warranted to optimize exercise behavior change and impact clinical outcomes. This could include messages that better address each person’s unique exercise barriers, for example, use of the program at more distal time points when adherence substantially declines and symptomatic benefits are reduced and testing the program in a pragmatic setting where patients are less motivated to exercise at the outset. Our results also highlight the need for further research to better understand the nature of the relationship between exercise adherence and clinical outcomes.

### Conclusions

This study showed that a behavior change theory–informed SMS program increased self-reported adherence to unsupervised home exercise over 6 months, following an initial period of exercise supervised by a physiotherapist, in people with knee OA and obesity. However, greater exercise adherence did not translate into improvements in pain and function.

## Appendix

Multimedia Appendix 1 Exclusion criteria.

Multimedia Appendix 2 Description of the two exercise programs.

Multimedia Appendix 3 Description and frequency of behavior change messages included in the SMS intervention.

Multimedia Appendix 4 Description and frequency of logistic and other messages included in the SMS intervention.

Multimedia Appendix 5 SMS intervention message sequence including message interactions and triggers.

Multimedia Appendix 6 Baseline characteristics of participants who were missing at least one primary outcome and those who provided both primary outcomes.

Multimedia Appendix 7 Number (percentage) of participants with adverse events, co-interventions, or pain medication use during the 24 weeks.

Multimedia Appendix 8 Means (SD) at week 24 or mean (SD) change within groups, from baseline to week 24, and mean (95% CI) difference between groups (adjusted for baseline value of outcome, TARGET exercise group and dichotomized baseline adherence), for continuous outcomes, using complete case data.

Multimedia Appendix 9 Number (percentage) of participants reporting global improvement (adjusted for TARGET exercise group and dichotomized baseline adherence), using complete case data.

Multimedia Appendix 10 CONSORT E-HEALTH checklist (V 1.6.1).

## Abbreviations

AQoL: Assessment of Quality of Life instrument
EARS: Exercise Adherence Rating Scale
ICC: intraclass correlation coefficient
NRS: numeric rating scale
OA: osteoarthritis
RCT: randomized controlled trial
