# Supplementary material for: Behavior Change Text Messages for Home Exercise Adherence in Knee Osteoarthritis: Randomized Trial
Source: J Med Internet Res. 2020 Sep 28;22(9):e21749. doi: 10.2196/21749 (PMC7551110; doi:10.2196/21749)
Supplement: Multimedia Appendix 1 [file jmir_v22i9e21749_app1.docx]

**Multimedia Appendix 1:** Exclusion criteria

| i) Lateral ≥ medial joint space narrowing on x-ray;  ii) Knee surgery/joint injection in past 6 months or planned surgery in next 9 months;  iii) Current or past (4 weeks) oral corticosteroids use;  iv) Systemic arthritic conditions;  v) Past knee fracture or malignancy;  vi) Past hip/knee joint replacement/tibial osteotomy;  vii) Other condition affecting lower limb function;  viii) Participation in knee strengthening or neuromuscular/functional exercise in past 6 months or planning to start exercise in next 9 months;  ix) Unable to walk unaided;  x) Unable to commit to study requirements. |
| --- |
