# Supplementary material for: Behavior Change Text Messages for Home Exercise Adherence in Knee Osteoarthritis: Randomized Trial
Source: J Med Internet Res. 2020 Sep 28;22(9):e21749. doi: 10.2196/21749 (PMC7551110; doi:10.2196/21749)
Supplement: Multimedia Appendix 2 [file jmir_v22i9e21749_app2.docx]

**Multimedia Appendix 2:** Description of the two exercise programs

| **Description †** | **Non weight bearing quadriceps strengthening exercise program** | **Weight bearing functional exercise program** |
| --- | --- | --- |
| **Type of exercises** | 5 exercises performed in sitting or supine aiming to strengthen quadriceps   1. Quads over a roll 2. Knee extension in sitting through range 3. Knee extension with hold at 30 degrees flexion 4. Straight leg raise 5. Short arch knee extension | Five exercises performed in weight bearing positions aiming to strengthen hip abductors, hip extensors, quadríceps and hamstrings   1. Forwards/backwards sliding 2. Sideways sliding 3. Standing hip wall push/crab walking 4. Wall squats/chair stands 5. Step ups/downs |
| **Frequency #** | 3 x per week | 3 x per week |
| **Dosage** | 3 sets of 10 repetitions | 3 sets of 10 repetitions except exercise #3 - 2 sets of 5 sec holds or 30 steps |
| **Resistance** | Applied via ankle cuff weight or resistance band | Body weight |
| **Intensity** | 10-repetition maximum weight or level of effort 5-8 out of 10 on modified Borg Rating of Perceived Exertion scale | Level of effort 5-8 out of 10 on modified Borg Rating of Perceived Exertion scale |
| **Progression** | Increasing weight or resistance band | Increasing body weight, changing stance surface, and/or varying the number of repetitions, direction, and speed of movement |

† In the TARGET trial, participants were randomised into one of the two exercise programs and continued that program during the ADHERE trial

# Exercises were performed 4 x per week in the TARGET trial and reduced to 3 x per week during the ADHERE trial
