# Supplementary material for: Behavior Change Text Messages for Home Exercise Adherence in Knee Osteoarthritis: Randomized Trial
Source: J Med Internet Res. 2020 Sep 28;22(9):e21749. doi: 10.2196/21749 (PMC7551110; doi:10.2196/21749)
Supplement: Multimedia Appendix 4 [file jmir_v22i9e21749_app4.docx]

**Multimedia Appendix** **4**. Description and frequency of logistic and other messages included in the SMS intervention

BCT = Behaviour change technique.

Original source: Nelligan RK, Hinman RS, Atkins L, Bennell KL. A Short Message Service Intervention to Support Adherence to Home-Based Strengthening Exercise for People With Knee Osteoarthritis: Intervention Design Applying the Behavior Change Wheel; JMIR Mhealth Uhealth 2019;7(10):e14619. URL: <https://mhealth.jmir.org/2019/10/e14619>. Reproduced under the terms of Creative Commons Attribution 4.0 license.
