# Supplementary material for: Behavior Change Text Messages for Home Exercise Adherence in Knee Osteoarthritis: Randomized Trial
Source: J Med Internet Res. 2020 Sep 28;22(9):e21749. doi: 10.2196/21749 (PMC7551110; doi:10.2196/21749)
Supplement: Multimedia Appendix 5 [file jmir_v22i9e21749_app5.docx]

**Appendix 5**: SMS intervention message sequence including message interactions and triggers

BCT=behaviour change technique

* message prompts selection of one barrier, from a pre-specified list, which best explains low exercise adherence. Barriers: forgot, too tired, knee hurts so can’t exercise, worried exercise is causing pain, exercise isn’t helping, boring, lack of time, life stress, and none apply

† a response is supported if it uses the keywords provided in the preceding message

^ message suggests participants re-try or contact program staff, if needed.

Modified from: Nelligan RK, Hinman RS, Atkins L, Bennell KL. A Short Message Service Intervention to Support Adherence to Home-Based Strengthening Exercise for People With Knee Osteoarthritis: Intervention Design Applying the Behavior Change Wheel; JMIR Mhealth Uhealth 2019;7(10):e14619. URL: <https://mhealth.jmir.org/2019/10/e14619>. Reproduced under the terms of Creative Commons Attribution 4.0 license.
