# Supplementary material for: Behavior Change Text Messages for Home Exercise Adherence in Knee Osteoarthritis: Randomized Trial
Source: J Med Internet Res. 2020 Sep 28;22(9):e21749. doi: 10.2196/21749 (PMC7551110; doi:10.2196/21749)
Supplement: Multimedia Appendix 6 [file jmir_v22i9e21749_app6.docx]

**Multimedia Appendix 6.** Baseline characteristics of participants who were missing at least one primary outcome and those who provided both primary outcomes

| **Characteristics** | **Missing at least one primary outcome (n=11)** | **Provided both primary outcomes (n=99)** | ***P*-value** |
| --- | --- | --- | --- |
| TARGET exercise group, n (%) |  |  | .44 |
| Non-weight bearing exercise | 7 (64%) | 51 (52%) |  |
| Weight bearing exercise | 4 (36%) | 48 (48%) |  |
| Age (years), mean (SD) | 61.7 (8.6) | 62.4 (6.5) | .76 |
| Female, n (%) | 9 (82%) | 65 (66%) | .28 |
| Height (m), mean (SD) | 1.6 (0.1) | 1.7 (0.1) | .46 |
| Body mass (kg), median (IQR) | 101.6 (88.0-116.1) | 99.6 (88.8-111.2) | .72 |
| Body mass index (kg/m^2^), median (IQR) | 36.9 (34.7-40.2) | 35.3 (32.5-40.0) | .38 |
| Radiographic disease severity KL grade, n (%) |  |  | .85 |
| 2 | 2 (18%) | 18 (18%) |  |
| 3 | 6 (55%) | 61 (62%) |  |
| 4 | 3 (27%) | 20 (20%) |  |
| Currently employed, n (%) | 6 (55%) | 61 (62%) | .65 |
| Symptom duration (years), median (IQR) | 3.5 (2.5-10.0) | 5.5 (3.0-10.0) | .23 |
| Unilateral symptoms, n (%) | 4 (36%) | 20 (20%) | .22 |

KL=Kellgren and Lawrence; SD=standard deviation; IQR=interquartile range
