# Supplementary material for: Behavior Change Text Messages for Home Exercise Adherence in Knee Osteoarthritis: Randomized Trial
Source: J Med Internet Res. 2020 Sep 28;22(9):e21749. doi: 10.2196/21749 (PMC7551110; doi:10.2196/21749)
Supplement: Multimedia Appendix 7 [file jmir_v22i9e21749_app7.docx]

**Multimedia Appendix 7.** Number (percentage) of participants with adverse events, co-interventions, or pain medication use during the 24 weeks

|  | **SMS (n=56)** | **Control (n=54)** | ***P*-value** |
| --- | --- | --- | --- |
| **Adverse events** |  |  |  |
| Any adverse event | 9 (16%) | 8 (15%) | .53 |
| Knee pain | 5 (9%) | 3 (6%) | .38 |
| Pain in other areas | 4 (7%) | 5 (9%) | .48 |
| **Co-interventions**^§^ |  |  |  |
| At least one treatment ^†^ | 28 (62%) | 31 (67%) | .38 |
| Hot/cold treatment | 22 (49%) | 12 (26%) | .02 |
| Shoe orthotics | 7 (16%) | 9 (20%) | .41 |
| Massage | 10 (22%) | 12 (26%) | .43 |
| Knee braces | 11 (24%) | 8 (17%) | .29 |
| Hydrotherapy | 7 (16%) | 8 (17%) | .52 |
| Manual therapy | 4 (9%) | 7 (15%) | .27 |
| Walking stick | 4 (9%) | 4 (9%) | .63 |
| Acupuncture | 3 (7%) | 0 (0%) | .12 |
| Arthroscopic surgery | 0 (0%) | 1 (2%) | .51 |
| TENS | 0 (0%) | 1 (2%) | .51 |
| Ultrasound | 2 (4%) | 2 (4%) | .68 |
| Injections | 1 (2%) | 0 (0%) | .50 |
| Total knee replacement | 0 (0%) | 3 (7%) | .13 |
| **Pain medications**^§^ |  |  |  |
| Any pain medication | 32 (71%) | 34 (74%) | .47 |
| Non-steroidal anti-inflammatories | 21 (47%) | 22 (48%) | .54 |
| Cyclooxygenase-2 inhibitors | 2 (4%) | 3 (7%) | .51 |
| Analgesia, paracetamol | 30 (67%) | 28 (61%) | .36 |
| Topical anti-inflammatories | 20 (44%) | 17 (37%) | .30 |
| Oral corticosteroids | 1 (2%) | 2 (4%) | .51 |
| Oral opioids | 1 (2%) | 1 (2%) | .75 |

^§^n=45 for SMS and n=46 for control

TENS=transcutaneous electrical nerve stimulation

^†^ numbers do not add up to total as participants could choose more than one
