# Supplementary material for: Behavior Change Text Messages for Home Exercise Adherence in Knee Osteoarthritis: Randomized Trial
Source: J Med Internet Res. 2020 Sep 28;22(9):e21749. doi: 10.2196/21749 (PMC7551110; doi:10.2196/21749)
Supplement: Multimedia Appendix 8 [file jmir_v22i9e21749_app8.docx]

**Multimedia Appendix 8:** Mean (SD) at week 24 or mean (SD) change within groups, from baseline to week 24, and mean (95% CI) difference between groups (adjusted for baseline value of outcome, TARGET exercise group and dichotomized baseline adherence), for continuous outcomes, using complete case data

|  | **SMS** | **Control** | **Mean difference**  **SMS-Control** ^†^  **(95% CI)** | ***P*-value** |
| --- | --- | --- | --- | --- |
|  | **Mean (SD) at week 24** | |  |  |
| **Primary outcomes** |  |  |  |  |
| Adherence to prescribed home exercise (EARS section B) ^‡^ | 16.3 (6.6) | 13.4 (7.1) | 2.5 (0.3, 4.8) | .03 |
| No. of days home exercises completed in past week ^*‡^ | 1.9 (1.2) | 1.3 (1.2) | 0.5 (0.1, 0.9) | .02 |
| **Secondary outcomes** |  |  |  |  |
| Adherence to home exercise thrice weekly (NRS) ^*‡^ | 6.0 (3.8) | 5.1 (3.7) | 0.9 (-0.5, 2.2) | .21 |
|  | **Mean (SD) change within group (baseline minus week 24)** | |  | |
| Overall average knee pain (NRS) ^‡^ | -0.5 (2.0) | 0.1 (2.0) | -0.4 (-1.1, 0.4) | .31 |
| Pain (KOOS) | 0.9 (13.2) | -3.1 (12.6) | 2.9 (-2.2, 8.1) | .26 |
| Other symptoms (KOOS) | -0.4 (12.9) | -2.6 (12.8) | 1.5 (-3.7, 6.7) | .57 |
| Function (KOOS) | 1.4 (15.9) | -1.8 (12.6) | 1.8 (-3.9, 7.5) | .55 |
| Sport and recreation (KOOS) | -2.4 (19.8) | -3.8 (18.0) | 2.2 (-5.3, 9.7) | .57 |
| Knee-related quality-of-life (KOOS) | -0.7 (18.3) | -2.3 (15.8) | 1.7 (-5.1, 8.5) | .62 |
| Health related quality of life (AQoL) | 0.02 (0.10) | 0.02 (0.12) | 0.00 (-0.04, 0.05) | .94 |
| Self-Efficacy- Pain (ASES) | -0.1 (2.0) | 0.3 (2.6) | -0.3 (-1.1, 0.5) | .48 |
| Self-Efficacy- Function (ASES) | 0.1 (1.5) | -0.6 (2.1) | 0.2 (-0.4, 0.8) | .56 |
| Self-Efficacy- Other (ASES) | -0.1 (1.6) | -0.3 (2.2) | 0.2 (-0.5, 0.9) | .52 |
| Kinesiophobia (BFOMS) ^‡^ | 0.4 (2.0) | 0.3 (3.1) | 0.1 (-0.9, 1.1) | .86 |
| Pain catastrophising (PCS) ^‡^ | -1.3 (6.4) | 2.0 (10.3) | -2.0 (-5.1, 1.1) | .21 |
| Physical activity (PASE) | -4.5 (79.0) | -3.0 (70.4) | -4.7 (-34.3, 24.9) | .76 |

EARS=Exercise Adherence Rating Scale (0-24; higher scores, better adherence); Adherence to home exercise thrice weekly: agreement with statement “I have been doing my exercise sessions 3 times each week as recommended” collected using an 11-point numeric rating scale (NRS) and terminal descriptors “strongly disagree”=0 to “strongly agree”=10; Overall average knee pain NRS (0-10; higher scores, worse pain); KOOS=Knee Injury and Osteoarthritis Outcome Score (0 to 100; lower scores, worse pain/symptoms/function/quality-of-life); AQoL=Assessment of Quality of Life instrument (-0.04-1.0; higher scores, better quality of life); ASES=Arthritis Self Efficacy Scale (1-10; higher scores, better efficacy); BFOMS=Brief Fear of Movement Scale (6-24; higher scores indicate greater fear). PCS=Pain Catastrophising Scale (0-52; higher scores, greater catastrophizing); PASE=Physical Activity Scale for the Elderly (0-400+; higher scores, greater activity).

^‡^For change within groups, positive changes indicate improvement. ^†^ For mean difference between groups, positive differences favour SMS

*Not adjusted for baseline value of outcome
