# Supplementary material for: Behavior Change Text Messages for Home Exercise Adherence in Knee Osteoarthritis: Randomized Trial
Source: J Med Internet Res. 2020 Sep 28;22(9):e21749. doi: 10.2196/21749 (PMC7551110; doi:10.2196/21749)
Supplement: Multimedia Appendix 9 [file jmir_v22i9e21749_app9.docx]

**Multimedia Appendix 9:** Number (percentage) of participants reporting global improvement (adjusted for TARGET exercise group and dichotomized baseline adherence), using complete case data.

|  | **SMS** | **Control** | **Odds Ratio (95% CI)** | **P-value** | **Relative Risk (95% CI)*** | ***P*-value** |
| --- | --- | --- | --- | --- | --- | --- |
| Improved overall† | 23/45 (51.11) | 19/46 (41.30) | 1.54 (0.64, 3.70) | 0.33 | 1.23 (0.71, 1.76) | .33 |

^†^Rated using 7-point scales with terminal descriptors of ‘much worse’ to ‘much better’, with those indicating ‘moderately better’ or ‘much better’ classified as improved.

* Relative risks >1 favour the intervention
